# Monte‐Carlo calculation of output correction factors for ionization chambers, solid‐state detectors, and EBT3 film in small fields of high‐energy photons

**DOI:** 10.1002/acm2.13753

**Published:** 2022-08-23

**Authors:** Vladimir A. Klimanov, Yury S. Kirpichev, Zarina K. Serikbekova, Alexandr V. Belousov, Grigorii A. Krusanov, Gonzalo Walwyn‐Salas, Vladimir N. Morozov, Maria A. Kolyvanova

**Affiliations:** ^1^ State Research Center—Burnazyan Federal Medical Biophysical Center Federal Medical Biological Agency of the Russian Federation Moscow Russia; ^2^ National Research Nuclear University MEPhI Moscow Russia; ^3^ Medscan LLC Moscow Russia; ^4^ Radiation Protection and Hygiene Center Havana Cuba; ^5^ Emanuel Institute of Biochemical Physics Russian Academy of Sciences Moscow Russia

**Keywords:** EGS software, output correction factor, radiotherapy, small‐field dosimetry

## Abstract

High‐energy accelerators are often used in oncological practice, but the information on the small‐field dosimetry for the photon beams with nominal energy above 10 MV is limited. The goal of the present work was to determine the values of the output correction factor ($k_{Q_{\mathrm{clin}},Q_{\mathrm{ref}}}^{f_{\mathrm{clin}},f_{\mathrm{ref}}}$) for solid‐state detectors (Diode E, PTW 60017; microDiamond, PTW 60019), EBT3 film, and ionization chambers (Semiflex, PTW 31010; Semiflex 3D, PTW 31021; PinPoint, PTW 31015; PinPoint 3D, PTW 31016) in the small fields formed by 10, 15, 18, and 20 MV photon beams. The output correction factors were calculated by Monte‐Carlo method using EGSnrc toolkit for six field sizes (from $0.5\times 0.5\;{\mathrm{cm}}^{2}$ to $10\times 10\;{\mathrm{cm}}^{2}$) for isocentric and constant source‐to‐surface distance (SSD) techniques. The decrease in the field size led to an increase in $k_{Q_{\mathrm{clin}},Q_{\mathrm{ref}}}^{f_{\mathrm{clin}},f_{\mathrm{ref}}}$ for ionization chambers, while for solid‐state detectors and radiochromic film, $k_{Q_{\mathrm{clin}},Q_{\mathrm{ref}}}^{f_{\mathrm{clin}},f_{\mathrm{ref}}}$ were less than unity at the smallest field size. A larger sensitive volume of ionization chamber corresponded to a stronger deviation of output correction factor from unity: 1.847 (125 mm^3^ PTW 31010) versus up to 1.183 (16 mm^3^ PTW 31016) at the smallest field of 10 MV beam. The calculated output correction factors were used to correct the output factors for PTW 60017, PTW 60019, and EBT3. The deviation of the corrected output factor from the results of Monte‐Carlo simulation did not exceed 3% in the fields from $1.0\times 1.0\;{\mathrm{cm}}^{2}$ to $4.0\times 4.0\;{\mathrm{cm}}^{2}$ for 10 and 18 MV beams. Thus, Diode E, microDiamond, and EBT3 film can be recommended for small‐field dosimetry of high‐energy photons.

## 1 INTRODUCTION

New technologies of external beam radiation therapy such as intensity‐modulated radiotherapy, volumetric‐modulated arc therapy, stereotactic radiosurgery, and stereotactic body radiotherapy (SBRT) have been intensively developed in the last two decades.^1^ In contrast to conventional radiotherapy, for which the standard procedures of absolute and relative dosimetry are established,^2^, ^3^ these techniques often used so‐called small fields.^4^ In essence, there are three physical conditions determining the assignment of photon beam to small fields (at least one needs to be fulfilled)^4^: (1) there is a loss of lateral charged particle equilibrium on the beam axis; (2) there is partial occlusion of the primary photon source by the collimating devices on the beam axis; (3) the size of the detector is similar or large compared to the beam dimensions. Small‐field dosimetry has several technical features: differences in photon energy spectra^5^, ^6^ and positioning accuracy of the detectors compared to traditional fields, absence of electronic equilibrium,^7^, ^8^, ^9^, ^10^ as well as volume averaging effect.^11^, ^12^, ^13^ In addition, placing the detector in a small field can cause a noticeable perturbation in the photon beam.^14^, ^15^ All these subtleties of small‐field dosimetry were first summarized by Alfonso et al.,^16^ and in 2017, International Atomic Energy Agency, together with American Association of Physicists in Medicine, published a detailed report TRS‐483 Code of Practice,^4^ containing the state of art of the dosimetry of static small fields for maximum photon beam energies up to 10 MV (mainly for 6 MV beams). At the same time, the information on the small‐field dosimetry for the photon energies above 10 MV is extremely limited, although medical accelerators generating bremsstrahlung photons with energies up to 20–24 MV could be used in oncological practice.^17^, ^18^ For example, the energies up to 18–20 MV are utilized in some cases in the dynamic conformal arc SBRT.^19^, ^20^

To measure the absorbed dose in small fields, it is important to take into account that each detector has its own individual characteristics, which may affect the dosimetry accuracy. For example, corrections for the dose profiles calculation as well as beam quality correction factors for the case of small fields of high bremsstrahlung energies were calculated in Refs.^21^, ^22^, ^23^, ^24^ In addition, the measured output factors for small fields require applying of correction factors for the ratio of the detector readings.^16^ Determination of output factors and output correction factors for photon energies ≥10 MV was performed in the works of Refs.^1^, ^25^, ^26^, ^27^ and Refs.,^28^, ^29^ respectively. In the present paper, the values of the output correction factor were determined in the small fields of 10, 15, 18, and 20 MV photon beams for a set of detectors widely used in clinical dosimetry: a diode detector Dosimetry Diode E (type 60017), a diamond detector microDiamond (type 60019), as well as an EBT3 dosimetric film, and four models of micro‐ionization chambers: a Semiflex Chamber (type 31010), a Semiflex 3D Chamber (type 31021), a PinPoint Chamber (type 31015), and a PinPoint 3D Chamber (type 31016).

## 2 MATERIALS AND METHODS

### 2.1 Output correction factor for detectors in small fields

The new formalism for small‐field dosimetry recommended in TRS‐483^4^ is based on the establishment of the new type of reference field for beam calibration, machine‐specific reference field (msr‐field), *f*_msr_. For most medical accelerators, the msr‐field matches with the standard reference field,$f_{\mathrm{msr}}\equiv f_{\mathrm{ref}}=10\times 10\;{\mathrm{cm}}^{2}$. For those accelerators, which cannot form a standard $10\times 10\;{\mathrm{cm}}^{2}$ reference field, the closest to $10\times 10\;{\mathrm{cm}}^{2}$ field is taken as the msr‐field. For relative dosimetry of small *f*_clin_‐beams, it is necessary to strictly apply the experimental definition of the output factor as the ratio of doses in *f*_clin_ and *f*_ref_ fields^4^:

(1) $$\Omega_{Q_{\mathrm{clin},}Q_{\mathrm{ref}}}^{f_{\mathrm{clin},}f_{\mathrm{ref}}}=\frac{D_{w,Q_{\mathrm{clin}}}^{f_{\mathrm{clin}}}}{D_{w,Q_{\mathrm{ref}}}^{f_{\mathrm{ref}}}}=\frac{M_{Q_{\mathrm{clin}}}^{f_{\mathrm{clin}}}}{M_{Q_{\mathrm{ref}}}^{f_{\mathrm{ref}}}}\;k_{Q_{\mathrm{clin},}Q_{\mathrm{ref}}}^{f_{\mathrm{clin},}f_{\mathrm{ref}}},$$

where $\Omega_{Q_{\mathrm{clin}},Q_{\mathrm{ref}}}^{f_{\mathrm{clin}},f_{\mathrm{ref}}}$ (or $\Omega_{Q_{\mathrm{clin}},Q_{\mathrm{msr}}}^{f_{\mathrm{clin}},f_{\mathrm{msr}}}$) is the output factor of the clinical field *f*_clin_ relative to the reference field *f*_ref_, *Q*_clin_ and *Q*_ref_ are the corresponding beam qualities. The ratio of absorbed doses $D_{w,Q_{\mathrm{clin}}}^{f_{\mathrm{clin}}}$ and $D_{w,Q_{\mathrm{ref}}}^{f_{\mathrm{ref}}}$ (“w” indicates the absorbed dose in water) in Equation (1) can be replaced by the ratio of the detector readings $M_{Q_{\mathrm{clin}}}^{f_{\mathrm{clin}}}$ and $M_{Q_{\mathrm{ref}}}^{f_{\mathrm{ref}}}$ multiplied by the so‐called output correction factor $k_{Q_{\mathrm{clin}},Q_{\mathrm{ref}}}^{f_{\mathrm{clin}},f_{\mathrm{ref}}}$, which converts the detector readings ratio to the absorbed dose ratio.

The output correction factor can be calculated using Monte‐Carlo method or measured experimentally.

If the reference $10\times 10\;{\mathrm{cm}}^{2}$ is taken as the msr‐field and assuming that in Equation (1), the integral detector response is proportional to the dose absorbed in this detector, the output correction factor can be calculated by Monte‐Carlo simulation using the international formalism by Alfonso et al.^16^:

(2) $$k_{Q_{\mathrm{clin},}Q_{\mathrm{ref}}}^{f_{\mathrm{clin},}f_{\mathrm{ref}}}=\frac{D_{w,Q_{\mathrm{clin}}}^{f_{\mathrm{clin}}}}{M_{w,Q_{\mathrm{clin}}}^{f_{\mathrm{clin}}}}/\frac{D_{w,Q_{\mathrm{ref}}}^{f_{\mathrm{ref}}}}{M_{w,Q_{\mathrm{ref}}}^{f_{\mathrm{ref}}}}≅\frac{D_{w,Q_{\mathrm{clin}}}^{f_{\mathrm{clin}}}}{D_{\det,Q_{\mathrm{clin}}}^{f_{\mathrm{clin}}}}/\frac{D_{w,Q_{\mathrm{ref}}}^{f_{\mathrm{ref}}}}{D_{\det,Q_{\mathrm{ref}}}^{f_{\mathrm{ref}}}},$$

where $D_{w,Q_{\mathrm{clin}}}^{f_{\mathrm{clin}}}$ and $D_{w,Q_{\mathrm{ref}}}^{f_{\mathrm{ref}}}$ stand for the average absorbed dose to water scored in a small voxel at the reference point in homogeneous water in a field of quality *Q*_clin_ and *Q*_ref_, respectively; $D_{\det,Q_{\mathrm{clin}}}^{f_{\mathrm{clin}}}$ and $D_{\det,Q_{\mathrm{ref}}}^{f_{\mathrm{ref}}}$ are the average doses scored in the sensitive volume of the investigated detector in a field of quality *Q*_clin_ and *Q*_ref_, respectively.

### 2.2 Monte‐Carlo calculation of phase space files

In the present paper, the output correction factors were calculated by Monte‐Carlo method using EGSnrc software toolkit.^30^ At the first step, the phase space files (PSFs) of the radiation field containing complete information on the characteristics of photons crossing virtual planes were calculated using BEAMnrc user code. The geometry of the head of Varian accelerators (valid for Novalis TX, Trilogy, Clinac iX, DX, C/D, EX, and cX), which included the main elements such as targets, primary collimator, flattening filters, mirror, ion camber, secondary collimator, was set in the input file BEAMnrc according to the package of data of the Varian High‐Energy Clinac machines.^31^ The baseline electron source parameterization was mono‐energetic with a circularly symmetric Gaussian FWHM=1mm (for 10 MeV) and FWHM=1.4mm (for 15, 18, and 20 MeV).^32^

The PSFs were generated on the surface of a water phantom at the distance of 90 and 100 cm from the accelerator's target, which corresponds to isocentric irradiation and irradiation with fixed solid‐state detector, respectively. The collimation jaws were set so that the radiation field sizes at d=10cm depth in the first case and on the surface of the water phantom in the second case were $0.5\times 0.5\;{\mathrm{cm}}^{2}$, $1.0\times 1.0\;{\mathrm{cm}}^{2}$, $2.0\times 2.0\;{\mathrm{cm}}^{2}$, $3.0\times 3.0\;{\mathrm{cm}}^{2},$ $4.0\times 4.0\;{\mathrm{cm}}^{2}$, and $10\times 10\;{\mathrm{cm}}^{2}$. The number of particles in each PSF was at least $2\times 10^{6}\;\mathrm{photons}/{\mathrm{cm}}^{2}$.

The following values of the parameters in BEAMnrc user code were used to simulate the radiation transport through the accelerator head (here and below they were selected empirically based on the recommendations presented in NRCC Report PIRS‐701^30^ to obtain optimal statistics and calculation time): electron and photon cutoffs ECUT=0.7MeV, PCUT=0.01MeV; maximum energy‐loss fraction per step ESTEPE = 0.25; energy threshold ESAVE = 2.0 MeV. To increase the number of bremsstrahlung, the technique of their directional splitting in the target was used with the parameter value NBRSPL = 2000 for 15 and 18 MV photon beams and NBRSPL = 500 for 10 and 20 MV beams.

### 2.3 Experimental verification of Monte‐Carlo calculation

The dose profiles (at d=10cm depth) and percentage depth doses (PDDs) were calculated using DOSXYZnrc toolkit and measured experimentally in the water phantom for different sizes of the small field at SSD=90cm. The PSFs calculated by the method described previously were used as the radiation sources for the calculation. The following values of the parameters in the user code were used: ECUT=0.7MeV, PCUT = 0.01 MeV, ESTEPE = 0.25, ESAVE = 2 MeV, and nsplit = 10. The absorbed dose was determined on a uniform grid in rectangular voxels with Δx=Δy values from 0.025 to 0.25 cm and Δz values from 0.1 to 0.25 cm depending on the field size. The stories number was selected so as to obtain a statistical uncertainty of no more than 1.0%.

**FIGURE 1:**
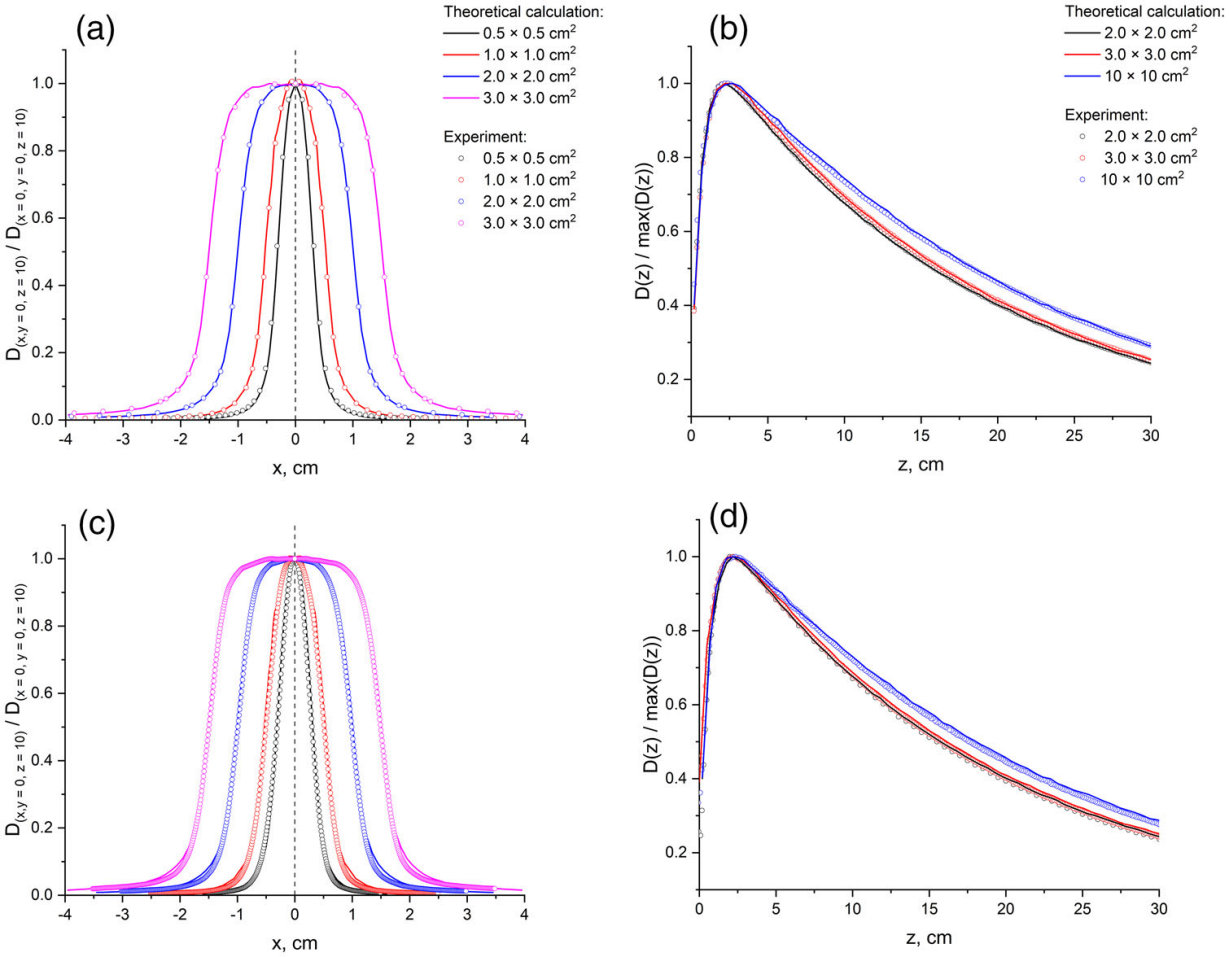
Comparison of dose profiles (a and c) and PDDs (b and d) for various field sizes calculated theoretically (lines) and determined experimentally (circles): (a and b) the data for microDiamond detector (c and d) the data for PFD^3G^ detector.

As an example, Figure 1a,c shows the results of the calculation of dose profiles and their experimental measurement using microDiamond and PFD^3G^ (IBA, Germany) detectors for $0.5\times 0.5\;{\mathrm{cm}}^{2}$, $1.0\times 1.0\;{\mathrm{cm}}^{2}$, $2.0\times 2.0\;{\mathrm{cm}}^{2}$, and $3.0\times 3.0\;{\mathrm{cm}}^{2}$ fields of 10 MV photon beam. Comparison of central‐axial dose distributions for $2.0\times 2.0\;{\mathrm{cm}}^{2}$, $3.0\times 3.0\;{\mathrm{cm}}^{2}$, and $10\times 10\;{\mathrm{cm}}^{2}$ fields, measured with the same detectors and simulated by Monte‐Carlo method, is presented in Figure 1b,d. It is clearly seen that the measured and simulated data are in good agreement. For both detectors, the discrepancy between theoretical and experimental dose profiles within the high‐dose region reached 5% for the smallest studied field, while for the other field sizes, it was less than 2%. In the penumbra region, the best agreement was found for the diamond detector. For PFD^3G^, the mean distance‐to‐agreement was ≤ 0.15 mm for all field sizes. When comparing the PDDs, the discrepancies in the readings of the studied detectors and the data of Monte‐Carlo simulation did not exceed 2%. So, the measured and simulated data are in good agreement. The verification of PSFs was also carried out for 18 and 20 MV beams (using Varian Clinac iX and Varian Trilogy, respectively). The results were generally similar in accuracy to the case of 10 MV (data not shown).

### 2.4 Calculation of equivalent square field size

The values of full width at half maximum (FWHM) for in‐line and cross‐line directions of the dose profile (FWHM*_x_* and FWHM*_y_*), as well as the equivalent square field size *S*_clin_ were determined in the water phantom at a 10 cm depth according to Ref.^4^ by Monte‐Carlo simulation. The *S*_clin_ values corresponding to different beam energies and nominal field sizes (*S*_col_) formed by the collimation jaws for SSD = 90 and 100 cm are presented in Table 1.

**TABLE 1:** Values of equivalent field size lengths (*S*_clin_).

	Field size (cm × cm)					
Energy (MV)	0.5 × 0.5	1.0 × 1.0	2.0 × 2.0	3.0 × 3.0	4.0 × 4.0	10 × 10
Isocentric technique (SSD = 90 cm, *d* = 10 cm)						
10	0.55	1.02	2.01	3.02	4.02	10.04
15	0.75	1.11	2.00	2.98	3.95	9.90
18	0.57	1.04	2.02	3.01	4.00	10.00
20	0.57	1.04	2.02	3.01	4.00	10.02
Constant SSD technique (SSD = 100 cm, *d* = 10 cm)						
10	0.61	1.12	2.2	3.31	4.34	10.9
15	0.85	1.21	2.19	3.26	4.33	10.8
18	0.63	1.13	2.14	3.30	4.4	11
20	0.63	1.13	2.13	3.31	4.4	11.1

### 2.5 Monte‐Carlo calculation of output correction factor

**FIGURE 2:**
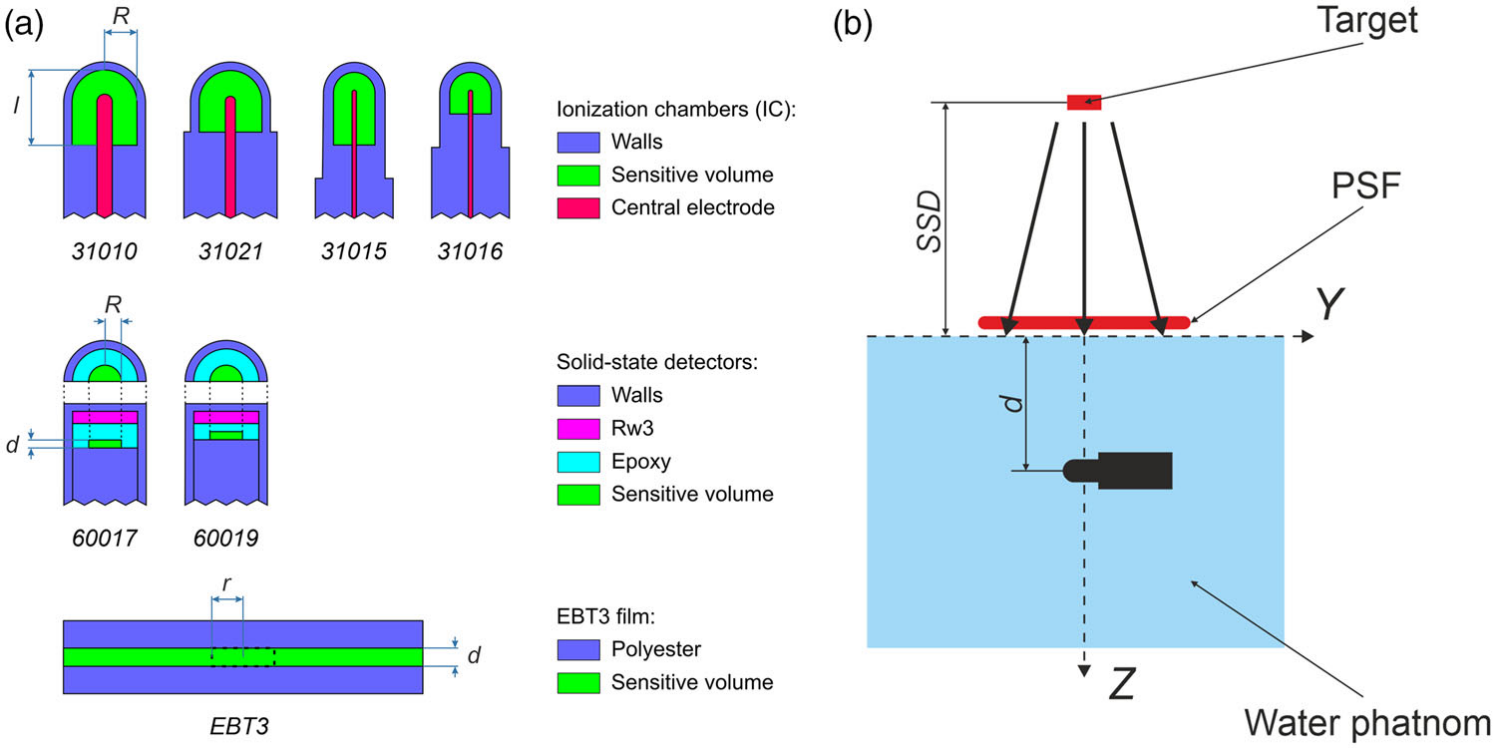
(a) Schematic representation (not in scale) of the studied ionization chambers, solid‐state detectors, and radiochromic film. (b) Orientation of the ionization chambers in the water phantom used in Monte‐Carlo simulation.

The output correction factors were calculated by Monte‐Carlo method using Equation (2) for two solid‐state detectors (Dosimetry Diode E, microDiamond) and four models of micro‐ionization chambers (Semiflex Chamber, Semiflex 3D Chamber, PinPoint Chamber, PinPoint 3D Chamber) manufactured by PTW (Germany), as well as for Gafchromic EBT3 film (Ashland, USA). The specification of PTW detectors were obtained from the manufacturer's manual,^33^ while the radiochromic film specification was taken from Ref.^34^ Their characteristics are presented in Table 2. The models of the detectors adapted from EGSnrc are schematically shown (not in scale) in Figure 2a. An insignificant simplification was made for simulation of the ionization chambers: the radii of the stems were taken equal to the radii of the chambers. Since the design of solid‐state detectors is much more complex,^35^ the simplifications were used for the simulation of their sensitive elements according to Refs.^36^, ^37^ The sensitive volumes were set as cylinders with dimensions and volumes specified in the manufacturer's manual. As it was noted by Francescon et al.,^38^ possible difference in the real size of the sensitive volume of microDiamond detector does not greatly affect the results of their simulation. The radiochromic film was set as 1 cm radius sample, and the dimensions of the active volume where the absorbed dose was calculated were r=0.1cm and d=30μm.

**TABLE 2:** Characteristics of the studied detectors (according to Refs.^33^, ^34^)

Detector	Sensitive volume material	Sensitive volume dimensions^a^	Sensitive volume walls/entrance window materials
Semiflex Chamber (type 31010)	Air	125 mm^3^ (*R* = 2.75 mm, *l* = 6.5 mm)	0.55 mm PMMA, 0.15 mm graphite
Semiflex 3D Chamber (type 31021)	Air	70 mm^3^ (*R* = 2.4 mm, *l* = 4.8 mm)	0.57 mm PMMA, 0.09 mm graphite
PinPoint Chamber (type 31015)	Air	30 mm^3^ (*R* = 1.45 mm, *l* = 5 mm)	0.57 mm PMMA, 0.09 mm graphite
PinPoint 3D Chamber (type 31016)	Air	16 mm^3^ (*R* = 1.45 mm, *l* = 2.9 mm)	0.57 mm PMMA, 0.09 mm graphite
microDiamond (type 60019)	C	0.004 mm^3^ (*R* = 1.1 mm, *d* = 0.001 mm)	0.3 mm RW3, 0.6 mm epoxy
Dosimetry Diode E (type 60017 Е)	Si	0.03 mm^3^ (*R* = 0.564 mm, *d* = 0.03 mm)	0.3 mm RW3, 0.4 mm epoxy
Radiochromic film (type EBT3)	H, Li, C, N, O, Na, Al, S, Cl	*d* = 0.03 mm	0.125 mm polyester

^a^ *d*: thickness, *l*: length, *R*: radius.

The detectors were placed in the $40\times 40\times 50\;{\mathrm{cm}}^{3}$ water phantom so that their reference points were located on a geometric axis of the beams. The axes of the ionization chambers were perpendicular to the beam axis (Figure 2b), and the reference points of the chambers were located at a depth of d=10cm. The solid‐state detectors were placed in parallel, and the radiochromic film was placed perpendicular to the beam axis so that the centers of their sensitive volumes were located at a 10 cm depth. To determine the absorbed dose in water, a cylinder of 0.2 cm height (*h*) and 0.2 cm radius (*r*) was set at a depth of 10 cm for $10\times 10\;{\mathrm{cm}}^{2}$ field. The cylinder of h=0.1cm and r=0.05cm was set for $0.5\times 0.5\;{\mathrm{cm}}^{2}$ field, and the cylinders of h=0.2cm and r=0.1cm were used for the other field sizes. Note that the larger sizes of cylinder were used for larger field sizes to improve the statistics. The absorbed dose was scored in rectangular voxels with a side of 0.1–0.4 cm depending on the field size, centered in the point of interest on the beam axis.

The absorbed doses in water and in the detectors were calculated using DOSXYZnrc and egs_chamber applications, respectively. The following parameters were used in the simulation: AE=AP=ECUT=ESAVE=10keV. The electron range rejection (ESAVE = 1.0 and 2.0 MeV) and the cross‐section enhancement (XCSE=32and64) in egs_chamber as well as NRCYCL = 10 in DOSXYZnrc were used to reduce a dispersion. To increase the number of photons passing through the sensitive volume of the detectors, a water shell of 1.0–1.5 cm thickness was distinguished around each detector in egs_chamber. The step value of the condensed path of electrons and positrons was set as ESTEPE = 0.25.

When calculating the absorbed dose for ionization chambers, the type A statistical uncertainty was ≤0.3%, for solid‐state detectors and radiochromic film, it was ≤(0.2–0.9)%. The statistical uncertainty was <0.2% when calculating the absorbed dose in water. Moreover, despite the known sensitivity of the output factor simulation results to the size of the electron focal spot and the geometry of the accelerator head,^39^, ^40^ a good agreement between calculated dose profiles and ones measured experimentally was established in our case.

### 2.6 Determination of output factors

The output factors for microDiamond, Dosimetry Diode E, PinPoint 3D Chamber, and EBT3 radiochromic film were determined experimentally for TrueBeam and Clinac IX accelerators (Varian Medical System, USA). The fields of $0.5\times 0.5\;{\mathrm{cm}}^{2}$, $1.0\times 1.0\;{\mathrm{cm}}^{2}$, $2.0\times 2.0\;{\mathrm{cm}}^{2}$, $4.0\times 4.0\;{\mathrm{cm}}^{2}$, and $10\times 10\;{\mathrm{cm}}^{2}$ were formed for SSD = 90 cm (the sizes of the fields were determined at SAD=100cm) using 10 MV (TrueBeam; TPR_20, 10_ = 0.74) and 18 MV (Clinac IX; TPR_20, 10_ = 0.784) photon beams with flattening filter. The $f_{\mathrm{ref}}=10\times 10\;{\mathrm{cm}}^{2}$ and $f_{\mathrm{col}}=4.0\times 4.0\;{\mathrm{cm}}^{2}$ fields were formed using the collimating jaws at both *X* and *Y* axes. The small fields $f_{\mathrm{col}}=1.0\times 1.0\;{\mathrm{cm}}^{2}$ and $f_{\mathrm{col}}=2.0\times 2.0\;{\mathrm{cm}}^{2}$ were formed using a multileaf collimator, wherein the collimating jaws were set at $2.0\times 2.0\;{\mathrm{cm}}^{2}$ and $5.0\times 5.0\;{\mathrm{cm}}^{2}$, respectively.

The output factors were obtained by Equation (1). For this, the average reading of the detectors by three measurements were taken. For all detectors except for the radiochromic film, the output factors were measured in a MP3 Water Phantom System (PTW) using Unidose E electrometer (PTW). The detectors were placed in the center of the beam based on the measurement of two orthogonal profiles. For solid‐state detectors, the voltage was set to 0 V in accordance with the manufacturer's recommendations, and for the ionization chamber, the voltage was of 400 V, respectively. No additional corrections for polarization and recombination coefficients were applied. For EBT3, the output factors were measured in polymethyl‐methacrylate (PMMA)/Solid Water phantoms at the same conditions. Before the measurements, the films were calibrated against the dose measured with 0.6 cm^3^ Farmer ionization chamber (type 30010; PTW) according to TRS‐398.^2^ The films were scanned using Expression 10000 XL scanner (Epson, Japan) within 24 h after exposure. Image processing was performed using ImageJ software according to the technique described in Ref.^41^

The output factors were also calculated for the case of water using Monte‐Carlo method. The values of $\Omega_{Q_{\mathrm{clin}},Q_{\mathrm{ref}}}^{f_{\mathrm{clin}},f_{\mathrm{ref}}}$ were determined as ratio of the doses in small water volumes for *f*_clin_ and *f*_ref_. The volumes were set as square cross‐section prisms with a side from 0.05 to 0.2 cm and a height from 0.2 to 0.5 cm depending on the field size and centered on the beams’ axis at a depth of 10 cm in the water phantom.

## 3 RESULTS

### 3.1 Monte‐Carlo calculation of output correction factor

The output correction factors for ionization chambers, solid‐state detectors, and radiochromic film upon exposure with 10, 15, 18, and 20 MV photon beams in different irradiation techniques calculated using Monte‐Carlo method are presented in Tables 3, 4, 5, 6, respectively. The values obtained for isocentric irradiation technique slightly varied from those for constant SSD technique due to the ≈10% difference in the equivalent field size length (see Table 1). The ionization chambers and the solid‐state detectors showed opposite dependencies of the output correction factor on the radiation field size. For the ionization chambers, the output correction factor increased with a decrease in the field size, while for diode, diamond detector, and radiochromic film, the $k_{Q_{\mathrm{clin}},Q_{\mathrm{ref}}}^{f_{\mathrm{clin}},f_{\mathrm{ref}}}$ decreased. For $4.0\times 4.0\;{\mathrm{cm}}^{2}$ radiation field, the calculated values of output correction factor were close to unity (<1%) for the entire set of studied detectors. At the same time, the deviation of $k_{Q_{\mathrm{clin}},Q_{\mathrm{ref}}}^{f_{\mathrm{clin}},f_{\mathrm{ref}}}$ from unity for solid‐state detectors and radiochromic film in small radiation fields was less than 4%, while the readings of ionization chambers differed more significantly. For $3.0\times 3.0\;{\mathrm{cm}}^{2}$ and $2.0\times 2.0\;{\mathrm{cm}}^{2}$ fields, the reading deviation was ≤4%, while for the fields less than $2.0\times 2.0\;{\mathrm{cm}}^{2}$, the deviation was up to ≈85%. The readings of the ionization chambers in small fields correlated with their sensitive volumes: a larger sensitive volume corresponded to a stronger deviation from unity with decreasing field size. For example, the highest calculated value of $k_{Q_{\mathrm{clin}},Q_{\mathrm{ref}}}^{f_{\mathrm{clin}},f_{\mathrm{ref}}}$ under 10 MV exposure for 125 mm^3^ Semiflex Chamber was 1.847, while for 16 mm^3^ PinPoint 3D Chamber, the highest value was 1.183. Since the detectors with the deviation of the output correction factor from unity larger than 5% are not recommended for clinical dosimetry,^4^ the obtained data prove some limitations of the studied ionization chambers for small‐field dosimetry in the high‐energy region.

**TABLE 3:** Calculated values of the output correction factor $k_{Q_{\mathrm{clin},}Q_{\mathrm{ref}}}^{f_{\mathrm{clin},}f_{\mathrm{ref}}}$ for the studied set of detectors for various irradiation techniques with 10 MV photon beam

	Nominal field size (cm × cm)					
Detector	0.5 × 0.5	1.0 × 1.0	2.0 × 2.0	3.0 × 3.0	4.0 × 4.0	10 × 10
Isocentric technique (SSD = 90 cm, *d* = 10 cm)						
Semiflex Chamber (type 31010)	1.847 (±0.41%)	1.161 (±0.35%)	1.028 (±0.31%)	1.013 (±0.31%)	1.000 (±0.55%)	1.000
Semiflex 3D Chamber (type 31021)	1.529 (±0.45%)	1.089 (±0.38%)	1.015 (±0.47%)	1.010 (±0.49%)	1.001 (±0.39%)	1.000
PinPoint Chamber (type 31015)	1.382 (±0.54%)	1.076 (±0.47%)	1.013 (±0.64%)	1.002 (±1.13%)	1.001 (±0.53%)	1.000
PinPoint 3D Chamber (type 31016)	1.183 (±1.45%)	1.049 (±1.44%)	1.012 (±1.48%)	1.005 (±1.56%)	1.002 (±1.54%)	1.000
microDiamond (type 60019)	0.978 (±1.66%)	0.982 (±1.68%)	0.989 (±1.72%)	0.995 (±1.77%)	0.999 (±1.83%)	1.000
Dosimetry Diode E (type 60017 Е)	0.968 (±1.59%)	0.986 (±1.59%)	1.003 (±1.60%)	1.007 (±1.73%)	1.002 (±1.67%)	1.000
Radiochromic film (type EBT3)	0.977 (±0.53%)	0.985 (±0.58%)	0.992 (±0.59%)	0.997 (±0.50%)	0.999 (±0.71%)	1.000
Constant SSD technique (SSD = 100 cm, *d* = 10 cm)						
Semiflex Chamber (type 31010)	1.685 (±0.75%)	1.137 (±0.71%)	1.016 (±0.73%)	1.007 (±0.74%)	1.001 (±0.74%)	1.000
Semiflex 3D Chamber (type 31021)	1.426 (±0.81%)	1.099 (±0.85%)	1.012 (±0.93%)	1.006 (±0.81%)	1.000 (±0.81%)	1.000
PinPoint Chamber (type 31015)	1.394 (±0.93%)	1.070 (±0.85%)	1.009 (±0.80%)	1.004 (±0.81%)	1.000 (±0.81%)	1.000
PinPoint 3D Chamber (type 31016)	1.130 (±1.27%)	1.032 (±1.33%)	1.006 (±1.31%)	1.003 (±1.41%)	1.000 (±1.39%)	1.000
microDiamond (type 60019)	0.962 (±1.67%)	0.983 (±1.89%)	1.004 (±2.05%)	1.007 (±2.05%)	1.006 (±2.05%)	1.000
Dosimetry Diode E (type 60017 Е)	0.982 (±1.48%)	0.985 (±1.51%)	0.991 (±1.58%)	0.996 (±1.60%)	0.99 (±1.60%)	1.000
Radiochromic film (type EBT3)	0.977 (±1.31%)	0.989 (±1.00%)	0.996 (±1.00%)	0.998 (±1.08%)	0.999 (±0.96%)	1.000

*Note*: The values of the relative standard uncertainty are presented in brackets.

**TABLE 4:** Calculated values of the output correction factor $k_{Q_{\mathrm{clin},}Q_{\mathrm{ref}}}^{f_{\mathrm{clin},}f_{\mathrm{ref}}}$ for the studied set of detectors for various irradiation techniques with 15 MV photon beam

	Nominal field size (cm × cm)					
Detector	0.5 × 0.5	1.0 × 1.0	2.0 × 2.0	3.0 × 3.0	4.0 × 4.0	10 × 10
Isocentric technique (SSD = 90 cm, *d* = 10 cm)						
Semiflex Chamber (type 31010)	1.690 (±1.04%)	1.200 (±1.09%)	1.033 (±1.00%)	1.020 (±1.03%)	1.002 (±1.06%)	1.000
Semiflex 3D Chamber (type 31021)	1.340 (±1.07%)	1.090 (±1.13%)	1.030 (±1.02%)	1.012 (±1.02%)	1.000 (±1.11%)	1.000
PinPoint Chamber (type 31015)	1.280 (±1.08%)	1.070 (±1.13%)	1.023 (±1.17%)	1.010 (±1.03%)	1.000 (±1.16%)	1.000
PinPoint 3D Chamber (type 31016)	1.124 (±1.46%)	1.040 (±1.56%)	1.008 (±1.39%)	1.000 (±1.38%)	1.000 (±1.38%)	1.000
microDiamond (type 60019)	0.975 (±2.40%)	0.987 (±2.44%)	0.995 (±2.49%)	0.998 (±2.50%)	0.999 (±2.50%)	1.000
Dosimetry Diode E (type 60017 Е)	0.970 (±0.76%)	0.989 (±1.04%)	1.007 (±1.13%)	1.008 (±1.12%)	1.005 (±1.02%)	1.000
Radiochromic film (type EBT3)	0.979 (±0.82%)	0.991 (±1.01%)	0.997 (±0.86%)	0.999 (±0.77%)	0.999 (±0.88%)	1.000
Constant SSD technique (SSD = 100 cm, *d* = 10 cm)						
Semiflex Chamber (type 31010)	1.534 (±0.42%)	1.149 (±0.54%)	1.032 (±0.47%)	1.010 (±0.48%)	1.003 (±0.42%)	1.000
Semiflex 3D Chamber (type 31021)	1.322 (±0.52%)	1.086 (±0.57%)	1.025 (±0.52%)	1.007 (±0.55%)	1.002 (±0.45%)	1.000
PinPoint Chamber (type 31015)	1.242 (±0.44%)	1.060 (±0.55%)	1.016 (±0.51%)	1.004 (±0.42%)	1.001 (±0.42%)	1.000
PinPoint 3D Chamber (type 31016)	1.123 (±0.98%)	1.050 (±0.97%)	1.004 (±0.87%)	1.001 (±0.88%)	1.000 (±0.86%)	1.000
microDiamond (type 60019)	0.992 (±1.33%)	0.993 (±1.47%)	0.995 (±1.42%)	0.996 (±1.48%)	0.997 (±1.45%)	1.000
Dosimetry Diode E (type 60017 Е)	0.980 (±1.58%)	0.994 (±1.60%)	1.002 (±1.49%)	1.006 (±1.53%)	1.002 (±1.57%)	1.000
Radiochromic film (type EBT3)	0.992 (±0.90%)	0.993 (±0.85%)	0.996 (±0.97%)	1.001 (±1.06%)	0.999 (±0.89%)	1.000

*Note*: The values of the relative standard uncertainty are presented in brackets.

**TABLE 5:** Calculated values of the output correction factor $k_{Q_{\mathrm{clin},}Q_{\mathrm{ref}}}^{f_{\mathrm{clin},}f_{\mathrm{ref}}}$ for the studied set of detectors for various irradiation techniques with 18 MV photon beam

	Nominal field size (cm × cm)					
Detector	0.5 × 0.5	1.0 × 1.0	2.0 × 2.0	3.0 × 3.0	4.0 × 4.0	10 × 10
Isocentric technique (SSD = 90 cm, *d* = 10 cm)						
Semiflex Chamber (type 31010)	1.705 (±0.90%)	1.192 (±1.25%)	1.034 (±1.26%)	1.011 (±1.36%)	1.007 (±0.97%)	1.000
Semiflex 3D Chamber (type 31021)	1.342 (±1.16%)	1.082 (±1.17%)	1.025 (±1.30%)	1.004 (±1.42%)	1.002 (±1.06%)	1.000
PinPoint Chamber (type 31015)	1.290 (±1.60%)	1.049 (±1.90%)	1.007 (±2.00%)	1.002 (±2.00%)	1.001 (±1.60%)	1.000
PinPoint 3D Chamber (type 31016)	1.139 (±1.60%)	1.033 (±1.90%)	1.011 (±1.90%)	1.002 (±1.93%)	1.001 (±1.54%)	1.000
microDiamond (type 60019)	0.963 (±1.37%)	0.982 (±1.52%)	1.002 (±1.78%)	1.003 (±1.89%)	1.002 (±1.61%)	1.000
Dosimetry Diode E (type 60017 Е)	0.967 (±2.01%)	0.994 (±2.10%)	1.003 (±2.20%)	1.005 (±2.28%)	1.003 (±2.00%)	1.000
Radiochromic film (type EBT3)	0.975 (±0.04%)	0.983 (±0.65%)	1.000 (±0.66%)	1.003 (±0.67%)	1.002 (±0.59%)	1.000
Constant SSD technique (SSD = 100 cm, *d* = 10 cm)						
Semiflex Chamber (type 31010)	1.568 (±0.47%)	1.153 (±0.54%)	1.028 (±0.47%)	1.006 (±0.48%)	1.003 (±0.42%)	1.000
Semiflex 3D Chamber (type 31021)	1.308 (±0.52%)	1.076 (±0.57%)	1.016 (±0.52%)	1.003 (±0.55%)	1.000 (±0.45%)	1.000
PinPoint Chamber (type 31015)	1.243 (±0.44%)	1.063 (±0.55%)	1.008 (±0.52%)	1.001 (±0.42%)	1.000 (±0.42%)	1.000
PinPoint 3D Chamber (type 31016)	1.111 (±0.98%)	1.032 (±0.97%)	1.005 (±0.87%)	1.002 (±0.88%)	1.000 (±0.86%)	1.000
microDiamond (type 60019)	0.980 (±1.38%)	0.986 (±1.47%)	0.997 (±1.42%)	0.998 (±1.48%)	1.004 (±1.45%)	1.000
Dosimetry Diode E (type 60017 Е)	0.970 (±1.58%)	1.007 (±1.60%)	1.009 (±1.49%)	1.008 (±1.53%)	1.007 (±1.57%)	1.000
Radiochromic film (type EBT3)	0.980 (±1.03%)	0.989 (±1.36%)	0.999 (±0.84%)	1.001 (±1.02%)	1.000 (±0.97%)	1.000

*Note*: The values of the relative standard uncertainty are presented in brackets.

**TABLE 6:** Calculated values of the output correction factor $k_{Q_{\mathrm{clin},}Q_{\mathrm{ref}}}^{f_{\mathrm{clin},}f_{\mathrm{ref}}}$ for the studied set of detectors for various irradiation techniques with 20 MV photon beam

	Nominal field size (cm × cm)					
Detector	0.5 × 0.5	1.0 × 1.0	2.0 × 2.0	3.0 × 3.0	4.0 × 4.0	10 × 10
Isocentric technique (SSD = 90 cm, *d* = 10 cm)						
Semiflex Chamber (type 31010)	1.748 (±0.60%)	1.149 (±0.68%)	1.016 (±0.61%)	1.007 (±0.60%)	1.002 (±0.65%)	1.000
Semiflex 3D Chamber (type 31021)	1.477 (±0.72%)	1.126 (±0.81%)	1.015 (±0.74%)	1.008 (±0.71%)	1.002 (±0.75%)	1.000
PinPoint Chamber (type 31015)	1.351 (±0.64%)	1.095 (±0.69%)	1.012 (±0.66%)	1.005 (±0.66%)	1.000 (±0.66%)	1.000
PinPoint 3D Chamber (type 31016)	1.163 (±0.89%)	1.036 (±0.93%)	1.007 (±0.88%)	1.003 (±0.91%)	1.000 (±0.86%)	1.000
microDiamond (type 60019)	0.965 (±1.60%)	0.997 (±1.67%)	1.005 (±1.59%)	1.004 (±1.70%)	1.001 (±1.70%)	1.000
Dosimetry Diode E (type 60017 Е)	0.970 (±1.22%)	0.985 (±1.27%)	0.993 (±1.21%)	0.999 (±1.27%)	1.000 (±1.40%)	1.000
Radiochromic film (type EBT3)	0.980 (±1.31%)	0.987 (±1.00%)	0.992 (±1.00%)	0.997 (±1.08%)	0.999 (±0.96%)	1.000
Constant SSD technique (SSD = 100 cm, *d* = 10 cm)						
Semiflex Chamber (type 31010)	1.612 (±0.64%)	1.131 (±0.65%)	1.014 (±0.72%)	1.004 (±0.65%)	1.001 (±0.66%)	1.000
Semiflex 3D Chamber (type 31021)	1.371 (±0.73%)	1.100 (±0.73%)	1.012 (±0.76%)	1.004 (±0.73%)	1.000 (±0.78%)	1.000
PinPoint Chamber (type 31015)	1.344 (±0.74%)	1.084 (±0.73%)	1.009 (±0.74%)	1.001 (±0.74%)	1.000 (±0.73%)	1.000
PinPoint 3D Chamber (type 31016)	1.144 (±1.10%)	1.032 (±1.06%)	1.006 (±1.03%)	1.001 (±1.08%)	1.000 (±1.05%)	1.000
microDiamond (type 60019)	0.967 (±1.77%)	1.000 (±1.80%)	1.004 (±1.65%)	1.003 (±1.71%)	1.001 (±1.71%)	1.000
Dosimetry Diode E (type 60017 Е)	0.975 (±1.28%)	0.989 (±1.30%)	0.996 (±1.24%)	0.998 (±1.28%)	0.999 (±1.26%)	1.000
Radiochromic film (type EBT3)	0.981 (±0.89%)	0.991 (±0.85%)	0.995 (±0.98%)	0.997 (±1.06%)	0.999 (±0.89%)	1.000

*Note*: The values of the relative standard uncertainty are presented in brackets.

### 3.2 Experimental measurements of output factor of the clinical fields

As an example, comparison of corrected values of the output factors of microDiamond, Dosimetry Diode E, PinPoint 3D Chamber, and EBT3 film with Monte‐Carlo simulation for 10 and 18 MV photon beams are presented in Tables 7 and 8, respectively. We did not use other ionization chambers because according to the simulation results, they turned out to be unsuitable for dosimetry of small fields for the case of high‐energy photon beams. According to Equation (1), the ratios of the detectors’ readings in the clinical and reference fields were multiplied by the previously determined values of the output correction factor from Tables 3 and 5. For microDiamond, the deviation of the corrected values detector from the results of computer simulation did not exceed 1%–2%. The corrected values of the output factor for Dosimetry Diode E coincided with the results of Monte‐Carlo calculation within 2%–3%. The values matching with these data of 98%–99% were also found for EBT3 radiochromic film. The uncertainty of experimental and calculated data was ≈1.5%(1σ). The values of the output factors calculated by Monte‐Carlo method were in good agreement with the data obtained for Exradin A16 Ion Chamber (0.007 cm^3^ sensitive volume; Standard Imaging, USA) for the case of Varian machine in Ref.^1^ For 18 MV photon beam in both works, the output factors were equal and amounted 0.784 and 0.902 for $2.0\times 2.0\;{\mathrm{cm}}^{2}$ and $4.0\times 4.0\;{\mathrm{cm}}^{2}$ fields, respectively. For 10 MV beam, the values obtained in the present work turned out to be 2%–3% less: 0.789 versus 0.817 for $2.0\times 2.0\;{\mathrm{cm}}^{2}$ field and 0.879 versus 0.900 for $4.0\times 4.0\;{\mathrm{cm}}^{2}$ field, respectively.

**TABLE 7:** Corrected values of the output factor $\Omega_{Q_{\mathrm{clin},}Q_{\mathrm{ref}}}^{f_{\mathrm{clin},}f_{\mathrm{ref}}}$ for microDiamond, Dosimetry Diode E, PinPoint 3D Chamber, and radiochromic film exposure with 10 MV photons at SSD = 90 cm

	Nominal field size (cm × cm)				
Detector	0.5 × 0.5	1.0 × 1.0	2.0 × 2.0	3.0 × 3.0	4.0 × 4.0
Monte‐Carlo simulation	0.401 (±0.24%)	0.638 (±0.31%)	0.789 (±0.25%)	0.845 (±0.24%)	0.879 (±0.34%)
microDiamond (type 60019)	0.402 (±3.01%)	0.636 (±2.63%)	0.791 (±2.14%)	0.850 (±2.10%)	0.886 (±2.13%)
Dosimetry Diode E (type 60017 E)	0.414 (±3.54%)	0.646 (±2.52%)	0.793 (±2.00%)	0.851 (±1.51%)	0.879 (±1.84%)
PinPoint 3D Chamber (type 31016)	0,380 (±3.61%)	0.630 (±2.33%)	0.799 (±1.71%)	0.857 (±1.66%)	0.890 (±1.82%)
Radiochromic film (type EBT3)	0.410 (±0,70%)	0.646 (±0.25%)	0.787 (±0.54%)	0.831 (±0.41%)	0.885 (±0.30%)

*Note*: The values of the relative standard uncertainty are presented in brackets.

**TABLE 8:** Corrected values of the output factor $\Omega_{Q_{\mathrm{clin},}Q_{\mathrm{ref}}}^{f_{\mathrm{clin},}f_{\mathrm{ref}}}$ for microDiamond, Dosimetry Diode E, and radiochromic film exposure with 18 MV photons at SSD = 90 cm

	Nominal field size (cm × cm)			
Detector	1.0 × 1.0	2.0 × 2.0	4.0 × 4.0	10 × 10
Monte‐Carlo simulation	0.595 (±0.24%)	0.784 (±0.63%)	0.902 (±0.44%)	1.000
microDiamond (type 60019)	0.602 (±1.6%)	0.787 (±1.5%)	0.891 (±1.5%)	1.000 (±0.5%)
Dosimetry Diode E (type 60017 E)	0.614 (±2.01%)	0.788 (±2.10%)	0.886 (±2.22%)	1.000 (±0.53%)
Radiochromic film (type EBT3)	0.600 (±0.54%)	0.798 (±0.65%)	0.902 (±0.59%)	1.000 (±0.22%)

*Note*: The values of the relative standard uncertainty are presented in brackets.

## 4 DISCUSSION

The dependencies of $k_{Q_{\mathrm{clin}},Q_{\mathrm{ref}}}^{f_{\mathrm{clin}},f_{\mathrm{ref}}}$ on the size of the radiation field for the studied ionization chambers upon 10, 15, 18, and 20 MV irradiation were quite similar to the data for 10 MV beams from TRS‐483.^4^ At the same time, calculated values of the output correction factor for ≥10 MV photons significantly exceeded those obtained for 6 MV beams (especially for the fields less than $3.0\times 3.0\;{\mathrm{cm}}^{2}$). First, this is due to an increase in the size of the region of absence of transverse electronic equilibrium; second, due to the ionization chamber volume averaging effects; and moreover, due to an increase in the slope of the decay of the photon beam transverse profile and an increase in the radiation flux perturbations caused by the placing the detectors within the field.

As expected, the output correction factor for the field sizes less than $2.0\times 2.0\;{\mathrm{cm}}^{2}$ strongly depends on the sensitive volume of the ionization chambers. If, in accordance with TRS‐483, the difference of ≈5% is taken as the applicability limit of the ionization chamber for small‐field dosimetry,^4^ all studied ionization chambers can be used for the field sizes above $2.0\times 2.0\;{\mathrm{cm}}^{2}$ and PinPoint 3D for the field sizes above $1.0\times 1.0\;{\mathrm{cm}}^{2}$. These measurement results correspond to the recommendations of these ionization chambers manufacturer.^33^ In addition, for the case of 10 MV beam, the deviations of our results for Semiflex and PinPoint 3D Chambers from corresponding TRS‐483^4^ data ranged from 0.2% to 0.7%.

The dependence of the output correction factors on the field size for the solid‐state detectors and the radiochromic films differs significantly from that obtained for the ionization chambers. For the Dosimetry Diode E, this can be explained, first, by the volumetric effect due to the extremely small size of the sensitive volume; second, by the close to the absolute visibility of the radiation source from the sensitive volume; and third, by the material of the sensitive volume of the detector. An increase in the contribution of scattered radiation to the fluence caused by a decrease in the field size leads to a change in the ratio of the effective mass energy absorption coefficients of water and silicon. For Diode E, the largest discrepancy with TRS‐483^4^ equals to 3% was found for the smallest studied field of $0.5\times 0.5\;{\mathrm{cm}}^{2}$. For the other fields, the deviations were 0.3%–1.1%.

The EBT3 radiochromic film had the closest to unity values of the output correction factor in small fields among all the detectors under study. This was largely due to the closeness of the effective atomic number and density of the active layer of the film to those of water parameters. According to Butson et al.,^34^ the effective atomic number of the EBT3 active layer is $Z_{\mathrm{eff}}=7.46$, while effective atomic number of water is $Z_{\mathrm{eff}}=7.42$. In addition, the placing of the dosimetric film into water practically does not disturb the distribution of radiation fluxes due to its small thickness. According to the results of Casar et al.,^27^ the volume averaging coefficient of the dosimetric film for 6 and 10 MV photon beams is $k_{\mathrm{vol}}=1.002$.

There are very contradictory data in the literature for the case of a diamond detector. As noted in Ref.,^42^ in various publications, the agreement in the character of the dependence of $k_{Q_{\mathrm{clin}},Q_{\mathrm{ref}}}^{f_{\mathrm{clin}},f_{\mathrm{ref}}}$ on the field size for small radiation fields with a side of ≥1 cm is observed for ^60^Co, 6 and 10 MV beams, whereas for smaller field sizes, the data differ significantly. In Ref.,^27^ the publications on this topic were divided into three groups. In the first two groups, which included works with Monte‐Carlo calculations (numerical approach) and semi‐empirical approach (combines measurements and numerical calculations), an upward deviation of the values of the output correction factor from unity was found.^43^, ^44^ In the third group, which included experimental works (empirical approach), the values of $k_{Q_{\mathrm{clin},}Q_{\mathrm{ref}}}^{f_{\mathrm{clin},}f_{\mathrm{ref}}}$ were found less than unity, as in Refs.^28^, ^45^ The same result for a diamond detector was obtained for 6 and 10 MV beams in Ref.^27^ However, in the high‐energy region (>10 MV), this point is not studied yet.

In the present work, we observed a decrease in $k_{Q_{\mathrm{clin},}Q_{\mathrm{ref}}}^{f_{\mathrm{clin},}f_{\mathrm{ref}}}$ with decreasing field size for the diamond detector under 10 MV photon irradiation. The increase in the beam quality did not change the character of the dependence within the uncertainty of results. The values of output correction factor obtained for microDiamond in the present work match with the data by Casar et al.^27^ with an accuracy from 98.5% to 99.9% in case of 10 MV beam of Varian TrueBeam linac. And for case of Elekta Versa HD, the convergence of the data was about 97.3%–99.5% (except $0.5\times 0.5\;{\mathrm{cm}}^{2}$ field—in this case, the difference was ≈7.4%). For the photon beams ≥10 MV, the deviation in the values of the output correction factor from unity for a diamond detector in our case was ≈2%–4% for all field sizes. This correlates well with the data by Casar et al.^27^ for Varian TrueBeam, while the deviation for the case of Elekta Versa HD accelerator in their work reached ≈10% (for the smallest field). Thus, in absolute values, the output correction factors obtained in this work deviate by 0%–0.7% from the TRS‐483 data for 10 MV machines.^4^ Thus, although the atomic number of carbon ($Z_{C}=6$) is less than that of water, for studied photon energies, a higher dose response was observed relative to water. This effect may be caused by the fact that the secondary charged particles generated in the materials adjacent to the sensitive volume of the detector make the main contribution to ionization due to the extremely small sensitive volume of the diamond detector.

## 5 CONCLUSION

In the present work, the values of the output correction factor for a set of detectors widely used in clinical small‐field dosimetry were calculated by Monte‐Carlo method with use of EGSnrc software toolkit in the little studied region of photon energy above 10 MV. The obtained data demonstrate the limitations of the studied ionization chambers (Semiflex Chamber, PTW 31010; Semiflex 3D Chamber, PTW 31021; PinPoint Chamber, PTW 31015; PinPoint 3D Chamber, PTW 31016) for small‐field dosimetry in the high‐energy region. It is found that in small fields formed by 10–20 MV photons, the dependence of the output correction factors on the field size for solid‐state detectors and radiochromic film differs crucially from that for the ionization chambers. The deviation of the output correction factors from unity for solid‐state detectors and EBT3 film was less than 5%. Also, for solid‐state detectors and EBT3 film, the experimental output factors, adjusted by the calculated values of the output correction factors, were in good agreement with the results of Monte‐Carlo simulation within the uncertainty. Thus, Diode E (PTW 60017), microDiamond (PTW 60019), and EBT3 film have shown acceptable results and can be recommended to small‐field dosimetry. All studied ionization chambers can be used in a more limited range of the field size (above $2.0\times 2.0\;{\mathrm{cm}}^{2}$), and PinPoint 3D can be used for the field size above $1.0\times 1.0\;{\mathrm{cm}}^{2}$.

## CONFLICT OF INTEREST

The authors declare they have no conflicts of interest.

## AUTHOR CONTRIBUTIONS

*Conceptualization*: Vladimir A. Klimanov and Maria A. Kolyvanova. *Methodology*: Vladimir A. Klimanov. *Software*: Vladimir A. Klimanov, Zarina K. Serikbekova, Alexandr V. Belousov, and Grigorii A. Krusanov. *Validation*: Maria A. Kolyvanova, Yury S. Kirpichev, Gonzalo Walvyn‐Salas, and Vladimir N. Morozov. *Formal analysis*: Vladimir A. Klimanov, Alexandr V. Belousov, and Maria A. Kolyvanova. *Investigation*: Vladimir A. Klimanov, Yury S. Kirpichev, Zarina K. Serikbekova, Alexandr V. Belousov, Grigorii A. Krusanov, Gonzalo Walvyn‐Salas, Vladimir N. Morozov, and Maria A. Kolyvanova. *Resources*: Vladimir A. Klimanov, Vladimir N. Morozov, and Maria A. Kolyvanova. *Writing—original draft preparation*: Vladimir A. Klimanov, Vladimir N. Morozov, and Maria A. Kolyvanova. *Writing—review and editing*: Vladimir A. Klimanov, Alexandr V. Belousov, Vladimir N. Morozov, and Maria A. Kolyvanova. *Visualization*: Vladimir N. Morozov, Alexandr V. Belousov, and Maria A. Kolyvanova. *Supervision*: Maria A. Kolyvanova. *Project administration*: Vladimir A. Klimanov and Maria A. Kolyvanova. *Funding acquisition*: Vladimir A. Klimanov and Maria A. Kolyvanova.
